# Evaluation of the analytical performance of endocrine analytes using sigma metrics

**DOI:** 10.1002/jcla.23581

**Published:** 2020-09-20

**Authors:** Yanming Liu, Yue Cao, Xijun Liu, Liangyin Wu, Wencan Cai

**Affiliations:** ^1^ Department of Laboratory Medicine YueBei People’s Hospital Shaoguan China; ^2^ Guangdong Provincial Key Laboratory of Medical Molecular Diagnostics, Institute of Aging Research Guangdong Medical University Dongguan China; ^3^ Department of Medical Technology Medical College of Shaoguan University Shaoguan China

**Keywords:** corrective action, internal quality control, quality goal index, root cause analysis, sigma metrics

## Abstract

**Background:**

(a) To evaluate the clinical performance of endocrine analytes using the sigma metrics (σ) model. (b) To redesign quality control strategies for performance improvement.

**Methods:**

The sigma values of the analytes were initially evaluated based on the allowable total error (TEa), bias, and coefficient of variation (CV) at QC materials level 1 and 2 in March 2018. And then, the normalized QC performance decision charts, personalized QC rules, quality goal index (QGI) analysis, and root causes analysis (RCA) were performed based on the sigma values of the analytes. Finally, the sigma values were re‐evaluated in September 2018 after a series of targeted corrective actions.

**Results:**

Based on the initial sigma values, two analytes (FT3 and TSH) with σ > 6, only needed one QC rule (1_3S_) with N2 and R500 for QC management. On the other hand, seven analytes (FT4, TT4, CROT, E2, PRL, TESTO, and INS) with σ < 4 at one QC material level or both needed multiple rules (1_3S_/2_2S_/R_4S_/4_1S_/10_X_) with N6 and R10‐500 depending on different sigma values for QC management. Subsequently, detailed and comprehensive RCA and timely corrective actions were performed on all the analytes base on the QGI analysis. Compared with the initial sigma values, the re‐evaluated sigma metrics of all the analytes increased significantly.

**Conclusions:**

It was demonstrated that the combination of sigma metrics, QGI analysis, and RCA provided a useful evaluation system for the analytical performance of endocrine analytes.

## Graphical abstract

Sigma Metrics, as an effective analytical tool, had been widely used in the laboratory quality management and the clinical performance evaluation of analytes. However, few studies reported how to apply Sigma Metrics in the management of quality control and performance improvement of analytes. In this study, we comprehensively used the analytical tools of root cause analysis, quality goal index (QGI) to established a personalized statistical quality control strategy based on the sigma metrics of analytes and verified its effectiveness.

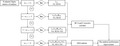

## 1 INTRODUCTION

In clinical laboratories, endocrine analytes are indicators of thyroid, pancreatic, and cortical function. They are frequently measured for diagnosis of diseases such as hyperthyroidism, hypothyroidism, diabetes, and Cushing's syndrome.^1^, ^2^, ^3^ Owing to their important role in the diagnosis and treatment of endocrine diseases, it is crucial to precisely and accurately evaluate their analytical performance.

The use of sigma (σ) metrics is a great success in the areas of customer satisfaction and global profitability,^4^ It was introduced into clinical laboratories by David Nevalainen1 in 2000.^5^ Currently, sigma metrics had been widely used in many aspects of laboratory quality management including pre‐analytic,^6^, ^7^ analytic,^8^, ^9^, ^10^ and post‐analytic ^11^ phases of testing. The analytical performance of analytes is quantitatively estimated as a sigma value. The value is calculated based on three parameters: allowable total error (TEa), bias, and coefficient of variation (CV).^11^ Though sigma metrics were applied for quality management of analytical biochemistry processes,^12^ it is rarely used for the quantitative immunoassay testing processes. This is particularly the case in testing the analytical processes of endocrine analytes.

In this study, the analytical performance of thirteen endocrine immunoassay analytes was evaluated by calculating their sigma values based on their TEa%, Bias%, and CV%. The quality control (QC) strategies were then personalized and redesigned for each analyte based on their sigma value. Moreover, the quality goal index (QGI) ratios of the analytes with σ below 4 were calculated to determine whether its precision or accuracy that needs to be improved first. Besides, the root cause analysis (RCA) and corrective actions were performed to reveal and eliminate the potential negative factors that affect analytical performance. Finally, the sigma metrics of the analytes were re‐evaluated to verify the validity of the RCA and corrective actions.

## 2 METHODS AND MATERIALS

### 2.1 Study design

**Figure 1:**
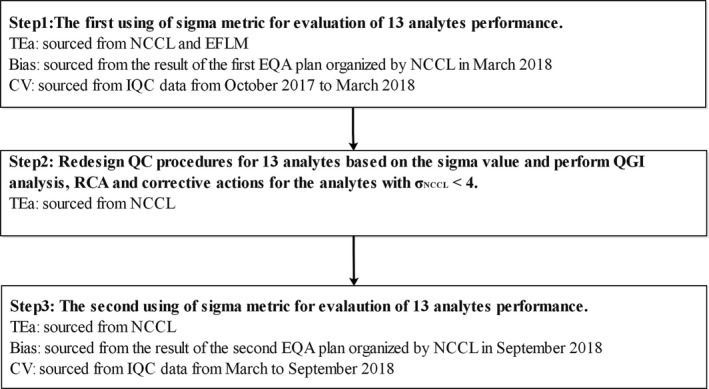
The research contents and roadmap of this present study

This study comprised three steps: the initial evaluation phase, RCA and corrective action step, and the re‐evaluation step (Figure 1). The study was conducted in the department of laboratory medicine of YueBei People's Hospital between October 2017 and September 2018. Sigma metrics values for the analytes were calculated using the following formula: σ=|TEa − Bias|/CV.^13^ This was the initial σ values of thirteen endocrine analytes. The QGI analysis, RCA, and corrective actions were performed, respectively, to find and eliminate the potential causes of poor clinical performance of the analytes. The σ values of the analytes were then re‐evaluated to verify the effectiveness of previous RCA and corrective activities.

### 2.2 Instrument, reagents, and analytes used

The analyzer of automatic electrochemical luminescent immunoassay analyzer (E602, Roche, Switzerland) and specific reagents were all purchased from Roche Inc. The endocrine analytes evaluated in this study were as follows: free triiodothyronine (FT3), triiodothyronine (TT3), free thyroxine (FT4), thyroxine (TT4), thyrotropin‐releasing hormone (TSH), cortisol (CROT), estradiol (E2), follicle stimulating hormone (FSH), luteinizing hormone (LH), progesterone (PROG), prolactin (PRL), testosterone (TESTO), and insulin (INS).

### 2.3 TEa

In this study, there were two sources of TEa as follows: one TEa was derived from the quality goals issued by the China National Center for Clinical Laboratories (NCCL) in 2017 throughout this research^5^; the other one, the minimum biological variation (BV) quality specification of TEa, was calculated based on the median of within‐subject (CV_I_) and between‐subject CV (CV_G_) from European Federation of Clinical Chemistry and Laboratory Medicine (EFLM) (https://biologicalvariation.eu/) (Table S1). According to the 2014 Milan strategic conference,^14^ the analytes’ TEa‐NCCL and TEa‐EFLM specifications, respectively, were constructed based on the effect of test performance on clinical outcomes and the components of biological variation of the measured.

### 2.4 Bias

In March and September 2018 (step 1 and step 3), two external quality assessment (EQA) plans of endocrine tests were successively organized by the NCCL in China. The five‐level materials of two EQA plans at varying concentrations of the analytes (LOT for the first EQA plan: 201811, 201812, 201813, 201814, and 201815; LOT for the second EQA plan: 201821, 201822, 201823, 201824, and 201825) were provided by the NCCL and assigned to our clinical laboratory in February 2018. Each level material was dissolved in pure water according to the manufacturer's instructions on the specific testing date specified by NCCL. The endocrine results of each level EQA material were then determined and reported to the NCCL in real time via an online portal. The bias of each material level of the analytes was then calculated using the determined value and the NCCL assigned value. In the process of RCA (step 2), the mean value, calculated by 2‐year accumulative bias of each analyte sourced from the NCCL EQA plans from 2016 to 2017, was used to calculate sigma value for exploring the effect of personnel and environmental factors on analytical performance. The calculation formula of bias was as follows,^15^ (taking the bias of FT3 as an example):

$${\text{Bias}}_{\text{FT3 level 1}}=\frac{\left|\text{FT}3_{\text{Level}\;1\;\text{determined value}}‐\text{FT}3_{\text{Level}\;1\;\text{assigned value}}\right|}{\text{FT}3_{\text{Level}\;1\;\text{assigned value}}}$$

$${\text{Bias}}_{\text{FT3}}=\frac{{\text{Bias}}_{\text{FT}3\;\text{Level}\;1}+{\text{Bias}}_{\text{FT}3\;\text{Level}2}+\ldots +{\text{Bias}}_{\text{FT}3\;\text{Level}5}}{5}$$

### 2.5 CV%

The daily internal quality control (QC) material Level 1 (LOT: 249 617, defined at a normal concentration) and Level 2 (LOT: 249 618, defined at an abnormal concentration) used in this study were purchased from Roche Inc. On one hand, the QC data (Level 1 and Level 2) collected between October 2017 and March 2018 were used to determine the cumulative CV of each analyte in the initial evaluation process of the σ value (step 1). Moreover, the QC data (Level 1 and Level 2) collected between March and September 2018 were used to determine the cumulative CV of each analyte in the re‐evaluation process of σ value (step 3). According to the national standard of China (Statistical interpretation of data‐Detection and treatment of outliers in the normal sample, GB/T 4883‐2018), the outliers were identified as the QC data out of the range (Mean ± 4 × Standard Deviation). After removing the outliers of QC data, both sets of data were analyzed using the DHC QC management software version 3.0. Noticeably, the mean and SD value of QC materials (Level 1 and Level 2) need re‐adjusted when the inspection conditions changed (such as different lot number of the reagents, equipment maintenance, and calibration). Correctly, the accumulated CV of any interested stage could be calculated by DHC QC management software.

### 2.6 QGI

QGI analysis helps laboratories to identify the main causes of low sigma value of analytes as well as excessive CV and bias or both.^16^, ^17^, ^18^ In this present study, the QGI ratios of the analytes with the initial σ_NCCL_ < 4 were calculated based on the formula QGI = Bias/(1.5 × CV).^19^ QGI < 0.8 indicates that the precision of the measurement procedure needs to be improved, QGI > 1.2 indicates that the accuracy of the measurement procedure needs to be improved, while 0.8 to 1.2 indicates that the precision and accuracy of the measurement procedure all need to be improved.

### 2.7 Construction of the normalized QC performance decision chart

**Figure 2:**
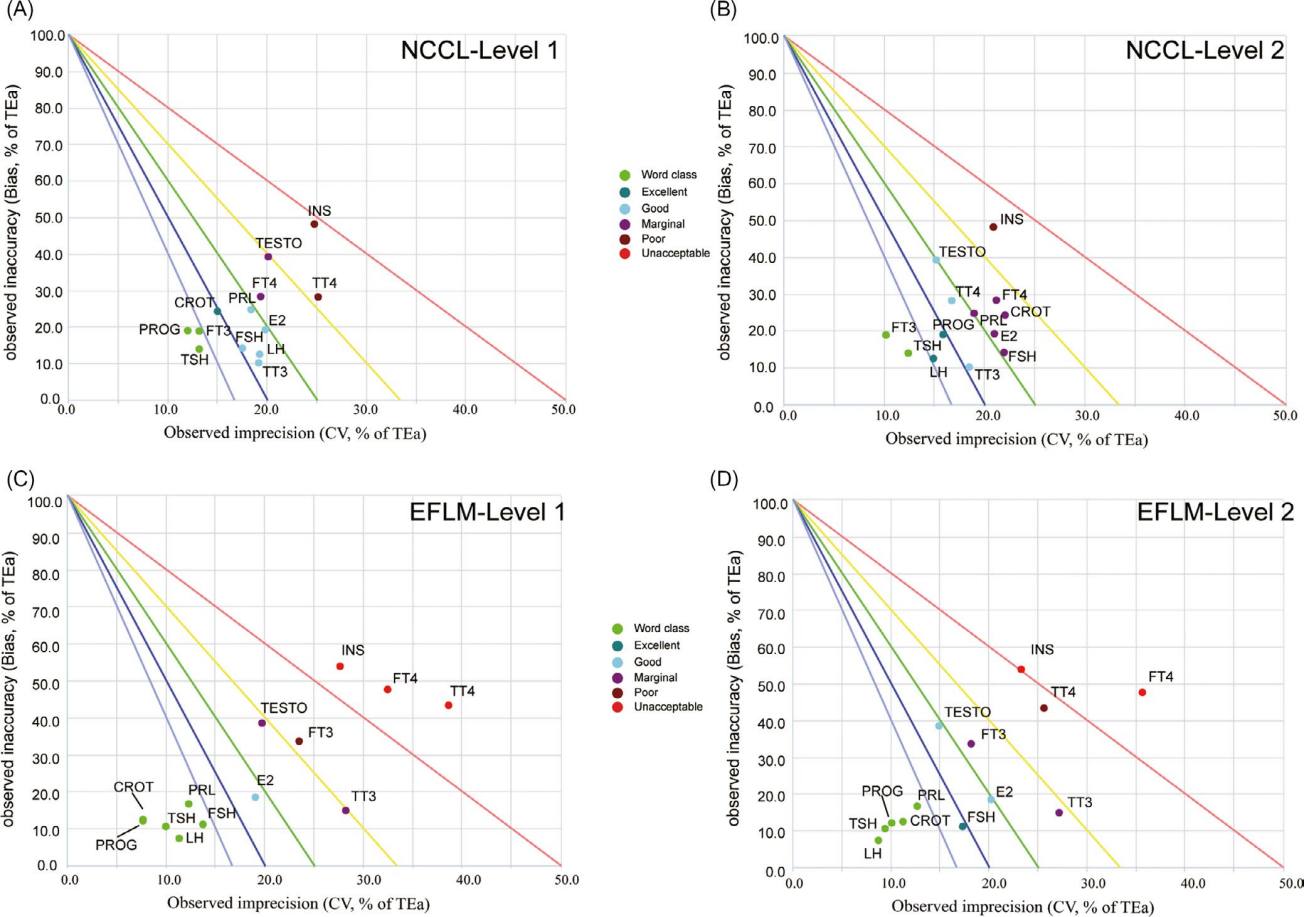
Normalized QC performance decision chart for 13 endocrine analytes (Levels 1 and 2) based on the first EQA plan of March 2018. (A) chart for QC material Level 1 (TEa‐NCCL). (B) chart for QC material Level 2 (TEa‐NCCL). (C) chart for QC material Level 1 (TEa‐EFLM). (D) chart for QC material Level 2 (TEa‐EFLM). The normalized performance decision diagram was drawn with CV/TEa as abscissa (imprecision) and Bias/TEa as ordinate (inaccuracy), and the chart is divided into six areas by five performance lines. Different colored circles represent different sigma grades

The normalized QC performance decision chart was constructed by registering an account in the CLInet (http://www.clinet.com.cn) with CV/TEa as abscissa and Bias/TEa as ordinate.^16^, ^17^, ^18^, ^20^, ^21^ The chart is divided into six grades by five lines.^22^ Based on the sigma level, the performance of the analytes was divided into six grades ^23^: world‐class (σ > 6), excellent (5 ≤ σ < 6), good (4 ≤ σ < 5), marginal (3 ≤ σ < 4), poor (2 ≤ σ < 3), and unacceptable (σ < 2) (Figure 2). The sigma value of the analyte was represented by colored circles marked in certain sigma grades of the chart when the parameters of the analyte's name, TEa, bias, and CV were inputted into the interface. This approach helped laboratory staff to obtain a visual synthesis view of the analytes’ performance in a single chart at each QC measurement level.

### 2.8 RCA and corrective activities

RCA was applied to determine the poor performance reasons for analytes with σ < 4.^15^, ^24^ It was performed based on five vital aspects: personal, equipment, material, method, and environment‐related to poor performance. This was done to determine multiple sources of poor performance rather than simply classifying an error as precision and/or an accuracy problem. Based on RCA results, appropriate improvement strategies were framed through brainstorming sessions with clinical quality management. The framed strategies were implemented for 6 months (from April to September 2018) in our clinical laboratory.

## 3 RESULTS

### 3.1 Initial sigma metrics evaluation of the analytes’ performance

The sigma metrics of every analyte at the QC material Levels 1 and 2 were calculated based on two kinds of TEa and summarized in Table 1. Normalized QC sigma charts were also constructed to visually evaluate the performance of the analytes at each QC material level (Figure 2). When we chose TEa‐NCCL for the sigma metric evaluation, nine of the thirteen analytes exhibited a performance of at least 4σ (good) at the QC material Level 1, and three of these analytes (FT3, TSH, and PROG) had a world‐class performance (Table 1 and Figure 2A). In the same line, seven of the thirteen analytes had a performance of at least 4σ (good) at NCCL Level 2. Two of these analytes (FT3 and TSH) had a world‐class performance (Table 1 and Figure 2B). The performance of two analytes (FT3 and TSH) was world‐class at both NCCL level 1 and 2 while that of nine analytes (FT4, TT4, CROT, E2, FSH, PRL, TESTO, and INS) exhibited σ < 4 at one or both QC material levels. Notably, INS had very poor analysis performance at both QC material levels (σ_NCCL_: 2.09 for Level 1 and 2.48 for Level 2). However, when we chose TEa‐EFLM for the sigma metric calculation, seven of the thirteen analytes exhibited a performance of at least 4σ (good) at QC material level 1, and five of these analytes (PROG, CROT, TSH, LH, PRL, and FSH) had a world‐class performance (Table 1 and Figure 2C). In the same line, eight of the thirteen analytes had a performance of at least 4σ (good) at EFLM Level 2. Five of these analytes (LH, TSH, PROG, CROT, and PRL) had a world‐class performance (Table 1 and Figure 2D). The performance of five analytes (PROG, CROT, TSH, PRL, and LH) was world‐class at both EFLM level 1 and 2 while that of six analytes (TESTO, FT3, TT3, INS, FT4, and TT4) exhibited σ < 4 at one or both QC material levels. Notably, TT4 and INS had very poor analysis performance at both QC material levels (σ_EFLM_ of TT4: 1.47 for Level 1 and 2.21 for Level 2; σ_EFLM_ of INS: 1.67 for Level 1 and 1.98 for Level 2). According to the performance evaluation results of analytes, it was found that different TEa sources (NCCL and EFLM) could perform a vital impact on the sigma metric calculation of analytes (Table 1). After a comprehensive analysis of the conformance between the analytes’ analytical performance and their clinical application, the TEa‐NCCL specifications were chosen for the calculation of sigma metrics in the process of QGI analysis, the QC strategies construction, RCA, and the re‐evaluation.

**Table 1:** Sigma metrics (Levels 1 and Level 2) for 13 endocrine analytes obtained from the Roche E602 calculated at the first EQA plan of March 2018

Analytes	TEa (%)		Bias (%)	CV (%)		Sigma metrics			
	TEa‐NCCL	TEa‐EFLM		Level 1	Level 2	Level 1		Level 2	
						σ_NCCL_	σ_EFLM_	σ_NCCL_	σ_EFLM_
FT3	25.00	14.01	4.71	3.29	2.55	6.17	2.83	7.97	3.65
TT3	25.00	17.03	2.53	4.80	4.62	4.68	3.02	4.86	3.14
FT4	25.00	14.88	7.08	4.83	5.30	3.71	1.61	3.38	1.47
TT4	20.00	13.02	5.64	5.03	3.34	2.86	1.47	4.29	2.21
TSH	25.00	33.04	3.48	3.30	3.10	6.53	8.96	6.95	9.54
CROT	25.00	48.80	6.07	3.76	5.51	5.04	11.36	3.44	7.75
E2	25.00	26.01	4.80	4.97	5.26	4.07	4.27	3.84	4.03
FSH	25.00	31.80	3.53	4.38	5.50	4.90	6.46	3.91	5.14
LH	25.00	42.59	3.12	4.82	3.74	4.54	8.19	5.85	10.55
PROG	25.00	39.32	4.74	3.01	3.96	6.73	11.49	5.11	8.73
PRL	25.00	37.20	6.19	4.59	4.73	4.10	6.76	3.98	6.56
TESTO	25.00	25.45	9.80	5.03	3.79	3.02	3.11	4.01	4.13
INS	25.00	22.35	12.04	6.19	5.22	2.09	1.67	2.48	1.98

Note

EFLM, European Federation of Clinical Chemistry and Laboratory Medicine, represents the minimum quality specification of TEa calculated by the median of CV_I_ and CV_G_ sourced from the biological variation database of EFLM; NCCL, the National Center For Clinical Laboratories, represents the TEa sourced from the quality goals issued by NCCL.

### 3.2 QC procedure redesigned for the analytes based on sigma metrics

The redesigned QC procedures for the thirteen analytes at different QC material levels are shown in Table 2. For analytes FT3 and TSH that had a “world‐class” analytical performance (σ ≥ 6) at both QC material levels, only one QC rule (1_3S_), one measurement at two QC material levels (N2) per QC event, and a run size of 500 clinical samples between adjacent QC events (R500) were adopted for QC management (Table 2). For analytes CROT, LH, and PROG that had “excellent” analysis performance (5 ≤ σ < 6) at one or both QC material levels, three multi‐rules (1_3S_/2_2S_/R_4S_) with N2 and R500 were adopted for QC management. For analytes TT3, TT4, E2, FSH, LH, PRL, and TESTO that had “good” analysis performance (4 ≤ σ < 5) at one or both QC material levels, four multi‐rules (1_3S_/2_2S_/R_4S_/4_1S_) with N4 and R200‐500 were adopted for QC management. For analytes FT4, TT4, CROT, E2, FSH, PRL, TESTO, and INS that had “marginal,” “poor,” or “unacceptable” performance (σ < 4) at one or both QC material levels, five multi‐rules (1_3S_/2_2S_/R_4S_/4_1S_/10_X_) with N6 and R10‐380 were adopted for QC management. Only the run size of TT4 was smaller than its average daily measurements at QC materials level 1. This suggested that two or more QC events could be performed per day at QC materials level 1 (Table 2). These results further suggested that the sigma metrics values could help in designing personalized QC procedures for the analytes at each QC material level.

**Table 2:** The personalized QC procedures selected for 13 endocrine analytes according to their initial σ_NCCL_

Analytes	σ_NCCL_		QC procedure		Average daily measurements
	Level 1	Level 2	Level 1	Level 2	
FT3	6.17	7.97	1_3S_ with N2 and R500 (Ped = 0.928; Pfr = 0.00)	1_3S_ with N2 and R500 (Ped = 0.928; Pfr = 0.00)	215
TT3	4.68	4.86	1_3S_/2_2S_/R_4S_/4_1S_ with N4 and R500 (Ped = 0.965; Pfr = 0.03)	1_3S_/2_2S_/R_4S_/4_1S_ with N4 and R500 (Ped = 0.965; Pfr = 0.03)	42
FT4	3.71	3.38	1_3S_/2_2S_/R_4S_/4_1S_/10_X_ with N6 and R200 (‐)	1_3S_/2_2S_/R_4S_/4_1S_/10_X_ with N6 and R90 (‐)^a^	215
TT4	2.86	4.29	1_3S_/2_2S_/R_4S_/4_1S_/10_X_ with N6 and R25 (‐)^a^	1_3S_/2_2S_/R_4S_/4_1S_ with N4 and R440 (Ped = 0.962; Pfr = 0.03)	42
TSH	6.53	6.95	1_3S_ with N2 and R500 (Ped = 0.928; Pfr = 0.00)	1_3S_ with N2 and R500 (Ped = 0.928; Pfr = 0.00)	215
CROT	5.04	3.44	1_3S_/2_2S_/R_4S_ with N2 and R500 (Ped = 0.946; Pfr = 0.01)	1_3S_/2_2S_/R_4S_/4_1S_/10_X_ with N6 and R100 (‐)^a^	5
E2	4.07	3.84	1_3S_/2_2S_/R_4S_/4_1S_ with N4 and R260 (Ped = 0.928; Pfr = 0.03)	1_3S_/2_2S_/R_4S_/4_1S_/10_X_ with N6 and R310 (‐)^a^	68
FSH	4.90	3.91	1_3S_/2_2S_/R_4S_/4_1S_ with N4 and R500 (Ped = 0.965; Pfr = 0.03)	1_3S_/2_2S_/R_4S_/4_1S_/10_X_ with N6 and R380 (‐)^a^	35
LH	4.54	5.85	1_3S_/2_2S_/R_4S_/4_1S_ with N4 and R500 (Ped = 0.965; Pfr = 0.03) ^a^	1_3S_/2_2S_/R_4S_ with N2 and R500 (Ped = 0.946; Pfr = 0.01)	35
PROG	6.73	5.11	1_3S_ with N2 and R500 (Ped = 0.928; Pfr = 0.00)	1_3S_/2_2S_/R_4S_ with N2 and R500 (Ped = 0.946; Pfr = 0.01) ^a^	68
PRL	4.10	3.98	1_3S_/2_2S_/R_4S_/4_1S_ with N4 and R260 (Ped = 0.928; Pfr = 0.03)	1_3S_/2_2S_/R_4S_/4_1S_/10_X_ with N6 and R460 (‐)^a^	35
TESTO	3.02	4.01	1_3S_/2_2S_/R_4S_/4_1S_/10_X_ with N6 and R30 (‐)^a^	1_3S_/2_2S_/R_4S_/4_1S_ with N4 and R200 (Ped = 0.920; Pfr = 0.03)	35
INS	2.09	2.48	1_3S_/2_2S_/R_4S_/4_1S_/10_X_ with N6 and R15 (‐)	1_3S_/2_2S_/R_4S_/4_1S_/10_X_ with N6 and R10 (‐)^a^	5

Note

The run sizes, Ped, and Pfr of QC procedures were estimated value based on this novel study.^17^, ^28^ The average daily measurements of analytes were sourced from statistical analysis of the total measurements in 2017. N: the total number of QC measurements per run of Roche E602, N2 represents two measurements at a single QC material level or one measurement at two QC material levels, similar definitions apply to N4 and N6. R: the run size of patient samples between QC events, R500 represents a run size of 500 patient samples between QC events. (‐): represent the Ped and Pfr of this QC procedure were not clear.

^a^ When the two levels of QC procedures of the same analyte were different, the more strict QC procedure (more rules, larger N, and smaller R) was selected for daily QC management.

### 3.3 QGI analysis, RCA, and corrective actions

**Figure 3:**
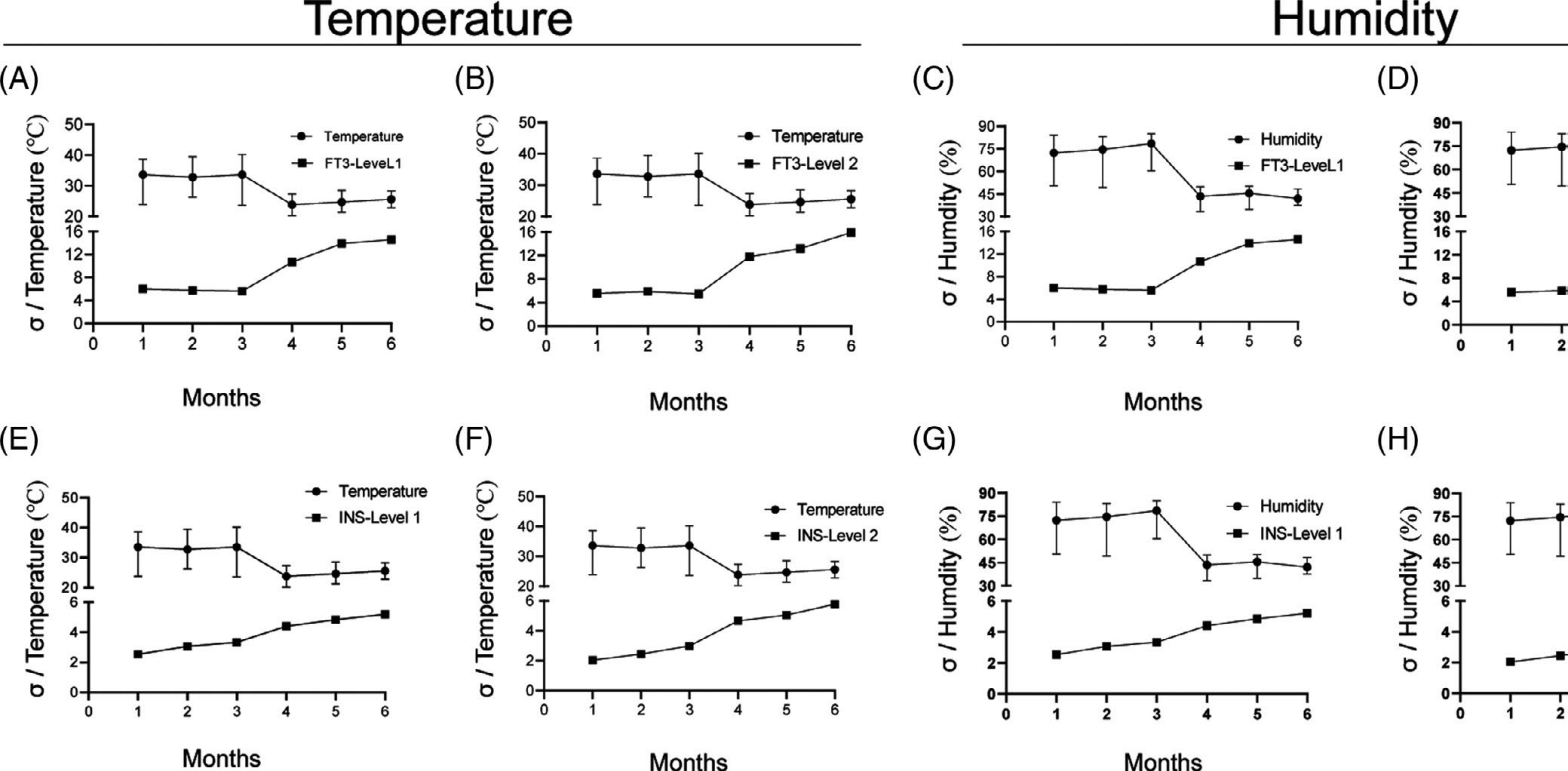
The effects of analytical temperature and humidity on the performance of analytes evaluated with sigma metrics (Level 1 and Level 2) by the same staff. The sigma value of analytes was calculated using the TEa‐NCCL. (A) temperature VS QC material level 1 of FT3; (B) temperature VS QC material level 2 of FT3; (C) humidity VS QC material level 1 of FT3; (D) humidity VS QC material level 2 of FT3; (E) temperature VS QC material level 1 of INS; (F) temperature VS QC material level 2 of INS; (G) humidity VS QC material level 1 of INS; (H) humidity VS QC material level 2 of INS. The temperature and humidity were automatically measured and acquired every half hour by temperature and humidity sensor per day. The mean temperature and humidity of the third hours after Roche E602 starting was represented as the temperature and humidity result today

The QGI analysis was thus performed to explore reasons for the low sigma metrics values. Four analytes (TT4, CROT, E2, and FSH) had poor precision at one QC material level, two analytes (FT4 and PRL) had undesired accuracy and precision at one or both QC materials levels, while two other analytes (TESTO and INS) exhibited low accuracy at one or both QC materials levels (Table 3). Five root causal factors, personnel, equipment, material, method, and environment, were scrutinized to identify the root causes of poor precision, accuracy or both (Table S2). For instance, four analytes (two analytes with σ_NCCL_ < 4 and two analytes with σ_NCC_L > 6) independently detected by two staff were evaluated using sigma metrics to explore the personnel factors (Table 4). Both staff had similar working conditions as well as similar QC material levels, brand reagents, and equipment. The sigma metrics values of the analytes (FT3 and TSH) that had world‐class performance (σ_NCCL_ > 6) investigated by staff A were significantly higher than those reported by staff B. This was also the case for the analytes (TESTO and INS) that had marginal or poor performance (σ_NCCL_ < 4). These results confirmed that the poor analysis performance of the analytes could be because of personnel problems. The operational skills of staff A were outstanding and while those of staff B were not appropriate. Cognizant to this, corrective actions that included relearning of the standard operation processes, operational skills retraining, and basic knowledge reassessment of all staff were performed to improve the quality of analysis (Table S2). Also, two analytes (FT3 with σ_NCCL_ > 6 and INS with σ_NCCL_ < 4) were analyzed by the same staff from January to June 2018 to evaluate the impact caused by the environmental factors (temperature and humidity) on the analysis performance. Based on the installation and stabilized operation of the new temperature and humidity control system in April 2018, the sigma metrics values of the two analytes increased (Figure 3). These findings demonstrated that the performance difference of these analytes was influenced by environmental changes. As such, environmental factors played an important role in analysis performance. Further to this, maintenance, reagents, and method factors were analyzed. Targeted measures were put in place to either eradicate or reduce the risk caused by these problems. The combined analysis of QGI analysis and RCA provided important strategies that helped in solving problems of poor analysis performance caused by existing or potential limiting factors.

**Table 3:** The QGI analysis of analytes with σ_NCCL_ < 4 at one or two QC materials levels

Analytes	σ_NCCL_		QGI		Problem
	Level 1	Level 2	Level 1	Level 2	
FT4	3.71	3.38	0.98	0.89	Precision and accuracy
TT4	2.86	4.29	0.75	^a^	Precision
CROT	5.04	3.44	^a^	0.73	Precision
E2	4.07	3.84	^a^	0.61	Precision
FSH	4.90	3.91	^a^	0.43	Precision
PRL	4.10	3.98	^a^	0.87	Precision and accuracy
TESTO	3.02	4.01	1.30	^a^	Accuracy
INS	2.09	2.48	1.30	1.54	Accuracy

^a^ Not applicable.

**Table 4:** The performance of four analytes evaluated with sigma metrics (Level 1 and Level 2) by two staff

Analytes	TEa‐NCCL (%)	Bias (%)^a^	staff A (20d)				staff B (20d)			
			Level 1		Level 2		Level 1		Level 2	
			CV (%)	σ_NCCL_	CV (%)	σ_NCCL_	CV (%)	σ_NCCL_	CV (%)	σ_NCCL_
FT3	25.00	4.25	2.56	8.11	2.20	9.43	3.60	5.76	3.45	6.01
TSH	25.00	3.68	2.34	9.11	2.12	10.06	4.20	5.08	3.87	5.51
TESTO	25.00	8.35	3.85	4.32	3.42	4.87	6.30	2.64	5.22	3.19
INS	25.00	11.50	4.00	3.38	4.20	3.21	5.19	2.60	5.84	2.31

Note

d: total number of days for QC measurements. Staff A: female, 28 years old, 8‐year seniority, technologist‐in‐charge, assigned by one company. Staff B: male, 23 years old, 1‐year seniority, technologist assigned by the other company.

^a^ The bias was the average value of 2‐year accumulative bias sourced from all the EQA plans issued by NCCL from 2016 to 2017.

### 3.4 Re‐evaluated analysis performance of the analytes in September 2018

**Figure 4:**
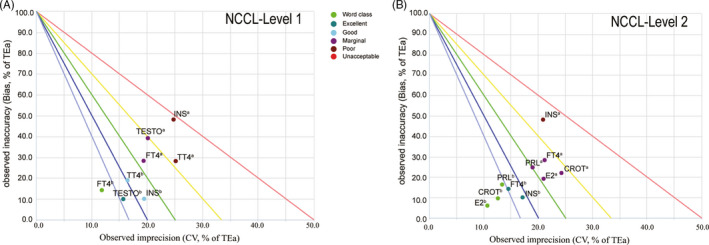
Significantly improved analytical performance of the analytes with the initial σ_NCCL_ < 4. The sigma value of analytes was calculated using the TEa‐NCCL. (A) Chart for QC material Level 1. (B) Chart for QC material Level 2. ^a^: the initial sigma value evaluated in March 2018; ^b^: the second sigma value re‐evaluated in September 2018

The sigma metrics of thirteen analytes were re‐evaluated in September 2018 after continuous RCA and corrective measures from April to September 2018. Based on the TEa sourced from NCCL, the performance of seven analytes (FT3, TT3, TSH, CROT, E2, LH, and PROG) reached the Six Sigma level (σ > 6) at both materials level (Table S3). Besides, three analytes (FT4, FSH, and PRL) exhibited a world‐class analysis performance at one QC material level (Table S3). The remaining three analytes also exhibited significantly improved sigma metrics values (σ > 4.6) at both QC materials level compared to the initial assessment results (Table S3). Circles of all the analytes that had initial sigma metrics values of less than 4 (σ < 4) moved down to the bottom left of the normalized QC performance decision chart (Figure 4). This was an indication that the bias and CV of these analytes had decreased with the improvement in precision and accuracy. Ultimately, the second sigma metrics evaluation results proved that the RCA and corrective actions performed were effective in improving the analysis performance of the analytes.

## 4 DISCUSSION

**Figure 5:**
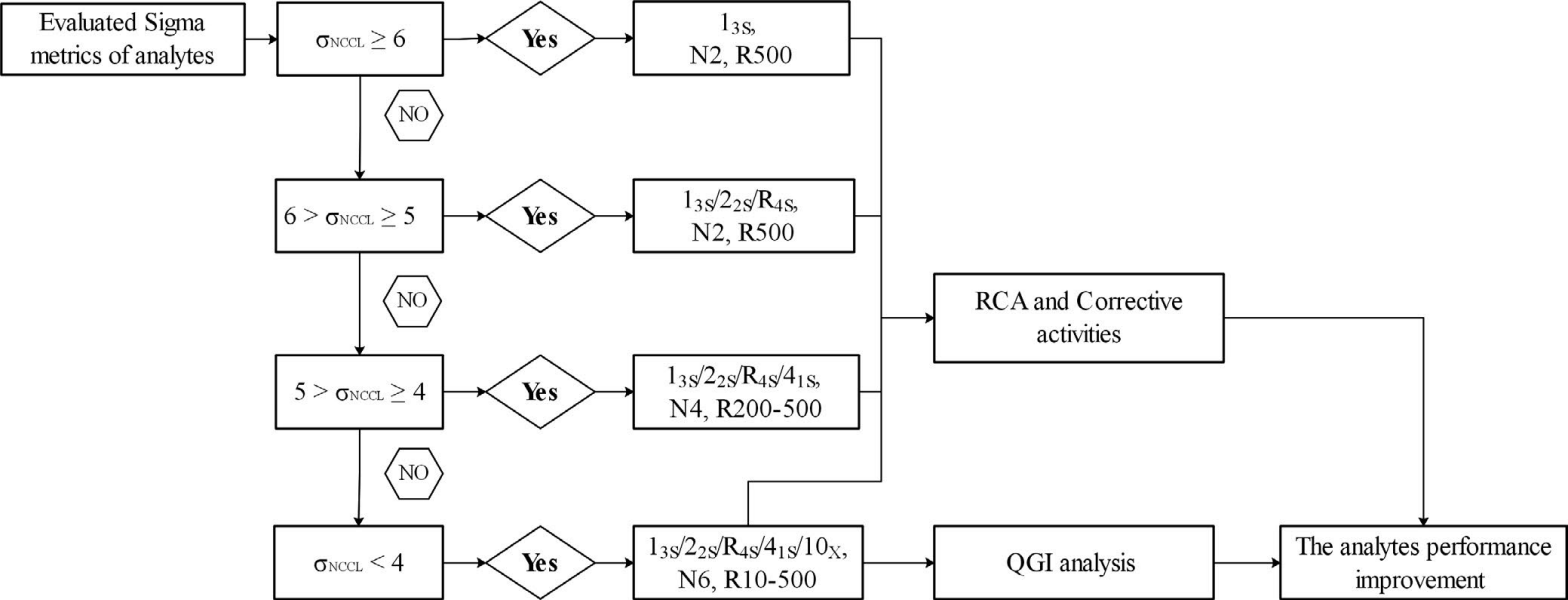
The workflow for performance improvement of endocrine analytes based on sigma metrics. The sigma value of analytes was calculated using the TEa‐NCCL. R, Run size of patient samples between QC events, R500 represents a run size of 500 patient samples between QC events; R200‐500 represents the run size interval from 200 to 500 depending on the sigma value of analytes, and a similar definition applied to R10‐500. When the two levels of QC procedures of the same analyte were different, the more strict QC procedure (more rules, larger N, and smaller R) was selected for analyte's performance improvement

Herein, the performances of thirteen endocrine analytes based on their sigma metrics values were analyzed. The initial sigma metrics values of the analytes were first evaluated in March 2018. The QC procedures for each analyte were then redesigned according to the academic theory of individualized QC and SQC based on their sigma metrics values. QGI analysis and RCA were then combined to further detect the inaccuracy and imprecision errors of the analytes with σ < 4. The targeted corrective actions were later implemented, and the analysis performance of the analytes re‐evaluated to verify the validity of the RCA and corrective actions. The sigma metrics value quality management workflow chart for the endocrine analytes in daily QC work was finally formulated and summarized (Figure 5). In clinical laboratories, sigma metrics had been widely used to evaluate and improve the quality of preanalytical, analytical, and postanalytical processes.^7^, ^25^, ^26^

According to our results, two important findings should be focused as follows: (a) the sigma metrics values difference of the same analyte at two QC materials levels; (b) the significant performance differences of the same analytes between TEa‐NCCL and TEa‐EFLM. These findings were consistent with those reported by Zhou B whose research in the practical application of sigma metrics management in analytical biochemistry processes.^15^ Zhou also reported that the factors of different detection systems, the sources of TEa, and the algorithms of CV and bias could be the common causes of this phenomenon.^15^ Moreover, acquiring the appropriate TEa is an important challenge while using sigma metrics for performance assessment. According to the TEa selection hierarchy of the European Federation of Clinical Chemistry and Laboratory Medicine (EFLM), there are three models (clinical outcomes, biological variabilities, and state‐of‐the‐art) to choose from when the required performance specifications were set in clinical laboratory.^27^ Importantly, the optimal TEa should be sourced from the establishment depending on the conditions of each clinical laboratory.^28^ Herein, the preferred TEa source was the quality goal released by NCCL for routine clinical immunization analytes in 2017. Another challenge was the source of Bias and CV. The bias sourced form the EQA program without metrological traceability. Moreover, the calculation of bias simply took the mean of five bias values from five‐level EQA materials without taking into account the effect the concentration of EQA materials on the estimated value of bias. Besides, the CV of level 1 and level 2 QC materials were the cumulative calculation of six months’ QC data. As such, the appropriate and reasonable algorithms for calculating bias and CV values were applied to calculate the sigma values for each analyte. This was done to reduce the impact of the source of bias and CV on the sigma value. Cognizant to this, analytical laboratories should consider the limitations of selecting TEa, bias, and CV calculation before using sigma metrics.

In our clinical laboratory, the QC procedure (2_2S_/1_3S_) empirically selected to supervise the analysis performance of all the analytes ran once per day to give independent measurements for the two QC material levels for all the analytes. Individualized QC and statistical QC (SQC) based on sigma metrics^13^, ^17^ were also introduced to improve the probability of error detection (Ped) and reduce the probability of false rejection (Pfr). Moreover, the Westgard Sigma rule, the total number of control measurements per QC event (N), and the run size of patient samples between two adjacent QC events (R) per day were also introduced in the SQC procedure of all the analytes. The selection of QC procedure is vital in balancing appropriate Pfr and Ped scores for the analytes as well as avoiding economic costs and overwork.^29^ For instance, based on the re‐evaluated sigma values of FT3, TT3, TSH, CROT, E2, LH, and PROG, the use of one QC rule (1_3S_) would result in a significant fall in the economic costs involved. The work efficiency would also greatly improve. However, there were two possible problems in the usage of QC procedures in this study. The adopted different QC strategies for a single analyte (TT4, CROT, E2, FSH, LH, PRL, and TESTO) in the initial evaluation of sigma metrics at different QC materials levels could result in low efficiency in daily work. Further to this, the shorter run size of analytes with σ < 4 (QC material level 1 of TT4) could result in more QC operation times per day. Interestingly, the run sizes for most analytes were generally greater than their average daily measurements. This indicated that the new QC strategies would not significantly increase the working intensity. Quality‐assured clinical outcomes and patient benefits would encourage more and more laboratories to choose targeted QC strategies to ensure the analytical performance of each analyte if the associated costs are reasonable.

Though the normalized QC performance decision chart provided visual performance differences of the analytes, it could not present the reasons for quality errors such as those caused by imprecision, inaccuracy, or both. This phenomenon was also observed by Qiu HW et al^22^ The QGI analysis was to remedy this defect by providing easy insights into where sigma quality improvement was required. Further to this, the RCA analysis provided a structural and standardized framework to investigate five potential causal factors (Table S2). This analysis also helped the laboratory staff to identify and efficiently solve the problems. However, the possible root causes were only established in the conditions of our laboratory (Table S2). As such, other superficial and deep‐seated problems could also exist. Based on the re‐evaluated sigma metrics, it was evident that solving the personnel and environmental factors improved the analysis performance of analytes that initially had low σ values. Based on these results, it was clear that personal continuous learning training, competent re‐evaluation, SOP documentation, extensive theoretical knowledge, and conscientiousness enhancement can help in improving the analysis quality of clinical laboratories. Besides, stable operations under the new temperature and humidity control system significantly minimized the high‐temperature and high‐humidity alarm of the Roche E602 analyzer thus providing an excellent analytical environment for performance analysis of the analytes. However, the sigma values of some analytes (FT4, TT4, FSH, PRL, TESTO, and INS) were still below six at one QC materials level or both even after QGI analysis, RCA, and corrective actions. This strongly suggested the existence of multiple root causes that were harder to discover and clear completely (especially the random error, such as needle blockage, problems with magnetic beads, incorrect use of controls, and poor lab water supply). Despite significant advances in clinical quality management strategies, the analysis performance of the analytes remained problematic.^30^

Nevertheless, there were four aspects of limitations in this research as follows: (a) Only the TEa‐NCCL of analytes were applied for the sigma metrics calculation in the analysis of QGI and personalized QC procedures. (b) The Ped and Pfr of the QC procedures (1_3S_/2_2S_/R_4S_/4_1S_/10_X_ with N6 and R) were not clear. (c) Some unknown factors caused the drift of the inspection system. (d) Further to this, three aspects of RCA, equipment maintenance, methods, and materials, were not assessed and thus their influence in the results was not reflected. In future studies, these aspects should be prioritized to generate more conclusive results.

## 5 CONCLUSION

The combination of sigma metrics evaluation, QGI analysis, RCA, corrective actions, and sigma re‐evaluation was adopted as a useful approach for performance improvement of analytes with σ < 4. Indeed, the sigma metrics method provided a useful evaluation system for the analytical performance of endocrine analytes.

## CONFLICT OF INTEREST

The authors stated that there are no conflicts of interest regarding the publication of this article.

## AUTHORS’ CONTRIBUTIONS

YL and YC designed the study, searched the literature, performed the experimental procedure, analyzed and interpreted the data, and wrote the study; XL, LW, WC performed the experimental procedure and searched the literature. All authors read and approved the final study.

## ETHICS APPROVAL AND CONSENT TO PARTICIPATE

Not applicable.

## CONSENT FOR PUBLICATION

All the authors agree on the publication of this article.

## Supporting information

Table S1

Table S2

Table S3
