# Supplementary material for: Evaluation of the analytical performance of endocrine analytes using sigma metrics
Source: J Clin Lab Anal. 2020 Sep 20;35(1):e23581. doi: 10.1002/jcla.23581 (PMC7843286; doi:10.1002/jcla.23581)
Supplement: Supplementary file 1 — Table S1 [file JCLA-35-e23581-s001.docx]

Supplementary file: Table S1. The desirable and minimum TEa of analytes sourced from EFLM

| Analytes |  | CV_I_^#^ |  | CV_G_^#^ |  | TEa (EFLM)* |
| --- | --- | --- | --- | --- | --- | --- |
| FT3 |  | 6.00 |  | 16.50 |  | 14.01 |
| TT3 |  | 9.20 |  | 11.90 |  | 17.03 |
| FT4 |  | 7.70 |  | 12.00 |  | 14.88 |
| TT4 |  | 6.40 |  | 12.00 |  | 13.02 |
| TSH |  | 15.90 |  | 31.90 |  | 33.04 |
| CROT |  | 23.10 |  | 48.70 |  | 48.80 |
| E2 |  | 15.00 |  | 13.00 |  | 26.01 |
| FSH |  | 12.40 |  | 42.10 |  | 31.80 |
| LH |  | 22.80 |  | 30.80 |  | 42.59 |
| PROG |  | 18.50 |  | 39.70 |  | 39.32 |
| PRL |  | 19.90 |  | 27.00 |  | 37.20 |
| TESTO |  | 13.00 |  | 21.30 |  | 25.45 |
| INS |  | 9.40 |  | 27.00 |  | 22.35 |

CV_B_: the biological variable coefficient; CV_I_: within-subject CV; CV_G_: between-subject CV; EFLM: European Federation of Clinical Chemistry and Laboratory Medicine (https://biologicalvariation.eu/). MQS: the minimum of TEa.

^#^: The analytes’ CV_I_ and CV_G_ were the median CV estimate sourced from EFLM (https://biologicalvariation.eu/).

^*^: The TEa-EFLM was the minimum biological variable quality specification of analytes’ TEa calculated based on the formula: TEa-(EFLM)=1.65×(0.75×CV_I_)+ 0.375×(CV_I_^2^+CV_G_^2^)^1/2^.
