# Supplementary material for: Evaluation of the analytical performance of endocrine analytes using sigma metrics
Source: J Clin Lab Anal. 2020 Sep 20;35(1):e23581. doi: 10.1002/jcla.23581 (PMC7843286; doi:10.1002/jcla.23581)
Supplement: Supplementary file 2 — Table S2 [file JCLA-35-e23581-s002.docx]

Supplementary file: Table S2. The RCA and corrective actions for the low performance of analytes

| **Aspects** | **RCA** | **Corrective actions** |
| --- | --- | --- |
| Personnel | ***Personnel factors associated with analysis performance***  1. weak conscientiousness attitude  2. poor theoretical knowledge  3. short seniority | ***Strengthen staff training as follows:***  1. Post responsibility and standard operating procedure (SOP) documents 2. Principle of electrochemical luminescence detection 3. Equipment (Roche E602) operation skills |
| Equipment (Roche E602) | ***Unstable performance of Roche E602*** 1. Insufficient and untimely equipment maintenance. 2. Blockage or crystallization of sample needle, reagent needle, and sipper needle. 3. Aging of measuring tank | ***Strengthen the maintenance and supervision of Roche E602*** 1. Strictly implement the maintenances of daily, weekly, two-week, and monthly 2. Clean each needle once per day before turning on and off 3. Focus on the results of daily APC check and replace the measuring tank in time if aging existing |
| Material | ***Unstable performance of the reagent kits*** 1.The reduced reagent performance caused by the problem of magnetic beads residing on the cap of the kit. 2. Incorrect recovery and reuse of the remaining reagents | ***Ensure the stable performance of reagent kits before use*** 1. Washing the residuary beads into the reagent before use 2. Do not recycle the residual reagent for detecting |
| Method | ***Incorrect preparation and testing of calibration and IQC materials*** 1. Improper dissolution of the materials of QC and calibration 2. Cross - use of QC materials with different batches 3. The parameters of the materials of QC and calibration are not updated timely | ***Perform preparation and testing of calibration and IQC materials normatively*** 1. The QC and calibration materials should be placed vertically for 15 minutes and upside down for 15 minutes after adding pure water, do not shake during the dissolving process. 2. Forbid staffs to crossly and alternately use the old and new batch of IQC materials. 3. The staff using the new batch number QC or calibration materials first is responsible for updating the corresponding parameters in the Cobas 8000 system timely. |
| Environment | ***Unstable environment factors*** 1. The unqualified conductivity of water source 2. The Unstable ambient temperature and humidity | ***Ensure environmental factors meeting the related requirements of Roche E602***  1. Check the operating status of the pure water equipment daily to ensure it's electrical conductivity less than 1μS/cm. 2. Install the temperature and humidity control system to ensure ambient temperature at 18-32℃，ambient humidity at 20-80%. |
