# Supplementary material for: Evaluation of the analytical performance of endocrine analytes using sigma metrics
Source: J Clin Lab Anal. 2020 Sep 20;35(1):e23581. doi: 10.1002/jcla.23581 (PMC7843286; doi:10.1002/jcla.23581)
Supplement: Supplementary file 3 — Table S3 [file JCLA-35-e23581-s003.docx]

Supplementary file: Table S3. Sigma metrics (Levels 1 and Level 2) for thirteen endocrine analytes reevaluated in September 2018

| Analytes |  | TEa-NCCL (%) |  | Bias (%) |  |  | CV (%) | |  | σ_NCCL_ | | |
| --- | --- | --- | --- | --- | --- | --- | --- | --- | --- | --- | --- | --- |
|  |  |  |  |  |  | Level 1 |  | Level 2 |  | Level 1 |  | Level 2 |
| FT3 |  | 25.00 |  | 2.09 |  | 1.88 |  | 1.48 |  | 12.21 |  | 15.53 |
| TT3 |  | 25.00 |  | 1.26 |  | 3.47 |  | 3.71 |  | 6.85 |  | 6.41 |
| FT4 |  | 25.00 |  | 3.56 |  | 2.96 |  | 3.62 |  | 7.24 |  | 5.92 |
| TT4 |  | 20.00 |  | 3.79 |  | 3.30 |  | 3.11 |  | 4.91 |  | 5.21 |
| TSH |  | 25.00 |  | 3.33 |  | 2.08 |  | 1.30 |  | 10.4 |  | 16.67 |
| CROT |  | 25.00 |  | 2.40 |  | 2.95 |  | 3.14 |  | 7.67 |  | 7.19 |
| E2 |  | 25.00 |  | 1.51 |  | 2.99 |  | 2.67 |  | 7.87 |  | 8.79 |
| FSH |  | 25.00 |  | 2.88 |  | 3.57 |  | 3.98 |  | 6.20 |  | 5.56 |
| LH |  | 25.00 |  | 2.31 |  | 3.72 |  | 3.74 |  | 6.10 |  | 6.07 |
| PROG |  | 25.00 |  | 1.59 |  | 3.67 |  | 3.54 |  | 6.37 |  | 6.61 |
| PRL |  | 25.00 |  | 4.09 |  | 3.51 |  | 3.34 |  | 5.97 |  | 6.27 |
| TESTO |  | 25.00 |  | 2.49 |  | 3.93 |  | 3.89 |  | 5.73 |  | 5.78 |
| INS |  | 25.00 |  | 2.52 |  | 4.87 |  | 4.28 |  | 4.62 |  | 5.25 |
